# Factors Influencing the Incidental Dose Distribution in Internal Mammary Nodes: A Comparative Study

**DOI:** 10.3389/fonc.2020.00456

**Published:** 2020-04-09

**Authors:** Wei Wang, Jinzhi Wang, Pengfei Qiu, Tao Sun, Yingjie Zhang, Qian Shao, Min Xu, Xijun Liu, Jianbin Li

**Affiliations:** ^1^Department of Radiation Oncology, Shandong Cancer Hospital and Institute, Shandong First Medical University and Shandong Academy of Medical Sciences, Jinan, China; ^2^Breast Cancer Center, Shandong Cancer Hospital and Institute, Shandong First Medical University and Shandong Academy of Medical Sciences, Jinan, China; ^3^Department of Medical Physics, Shandong Cancer Hospital and Institute, Shandong First Medical University and Shandong Academy of Medical Sciences, Jinan, China

**Keywords:** postmastectomy radiotherapy, internal mammary lymph node, incidental irradiation dose, anatomic parameters, technical parameters

## Abstract

**Objective:** To investigate the effect of anatomic and technical parameters on the incidental internal mammary lymph node (IMN) irradiation (IIMNI) dose among postmastectomy patients.

**Methods:** We retrospectively delineated the IMN on planning CT images from 138 patients who had undergone postmastectomy radiotherapy (PMRT). We analyzed the IIMNI dose coverage and its relationship with anatomic and technical parameters.

**Results:** The IIMNI mean dose was 32.85 ± 9.49 Gy, and 10 of 138 patients (7.25%) treated with PMRT received ≥45 Gy. In univariate analysis, the body weight, body mass index, body surface area, thoracic transverse diameter (D_T_), ratio of D_T_ to the thoracic anteroposterior diameter (D_AP_)(R_T/AP_), planning target volume of IMN (PTV_IMN_) included in PTV (IMNin) and the ratio of IMNin to PTV_IMN_ (R_IMNin_) and PTV posterior border were the parameters affecting IIMNI dose. In multivariate analysis, body weight, R_T/AP_, and R_IMNin_ were correlative factors that affected IIMNI dose.

**Conclusions:** For patients who underwent PMRT without IMN irradiation (IMNI), there was a wide variety in IIMNI doses. A minority of patients had adequate IIMNI dose coverage, and the higher IIMNI doses were associated with the less body weights and more R_IMNin_.

## Introduction

Irradiation of the chest wall and regional lymph nodes is considered the standard treatment strategy for breast cancer patients with positive axillary lymph nodes (ALNs) after mastectomy or modified radical mastectomy (MRM) due to its recognized success in improving local tumor control and overall survival (OS) (1–4). Internal mammary lymph nodes (IMNs) irradiation (IMNI) can improve disease-free survival (DFS) and OS in patients with breast cancer, and the mortality risk from ischemic heart disease with IMNI is equal to patients without IMNI (5, 6). However, in the Danish Breast Cancer Cooperative Group (DBCG)-IMN studies, patients with right-sided disease were allocated to the IMNI group, whereas left-sided patients were allocated to the no IMNI group (5). Therefore, potential bias might diminish the risk of radiation-induced heart disease. For the left-sided disease subset, one study that reviewed patients treated at a single institution from 1984 to 2007 showed no significantly increased hazard with IMN radiotherapy in planned three-dimensional conformal radiotherapy (3D-CRT) (7). Conversely, Taylor et al. reported a systematic review of heart doses from breast cancer radiation therapy studies published from 2003 to 2013 and concluded that IMNI doubled the typical heart dose (8). Long-term radiotherapy toxicity, especially heart toxicity, should be considered for patients with left-sided breast cancer in whom long-term survival is expected (9). In the contemporary irradiation techniques era, radiation-induced heart lesions are further lessened, and therapeutic drugs for heart and coronary artery disease are more diversified, so continued follow-up of cardiovascular disease induced by radiotherapy is still needed.

For patients with breast cancer (stage I, II, or III), observed IMN recurrence rate was <1% after primary breast cancer treatment, even when the IMNs are not excised or irradiated (6, 10). Patients most likely benefit from systemic therapies and incidental regional node irradiation (11, 12). According to current guidelines (e.g., NCCN), the internal mammary region should be included when regional lymph node irradiation is performed. It seems questionable whether a sub-group [that IMN receive high doses during incidental IMNI (IIMNI)] benefits from omission of IMNI, since high doses in the organs at risk (OARs) (heart and lung), comparable to IMNI, must be expected for these patients. Several studies have demonstrated the contribution of insufficient incidental radiation doses to IMNs using 3D-CRT (13–17). Some patients may not need IMNI, and this can minimize the risk of distant radiotherapy-induced cardiotoxicity, but there is insufficient evidence at this time to define such subgroups in detail. Individual risk assessment and optimization includes calculation of different treatment plans in high risk patients to evaluate the dose to internal mammary region.

So far, several studies have evaluated axillary dose levels using different techniques, and found dose distribution was directly influenced by the breast volume and shape (18–21). Active breathing control (ABC) technique can reduce the IMN coverage in left-sided breast cancer patients planned for postmastectomy radiation therapy (PMRT) (22). Both the dosimetric aspect and patient anatomy could eventually influence the regional lymph nodes of breast cancer. We postulated that patient anatomy and technical parameters are the potential factors influencing IIMNI dose variance, and aims to identify which patients receive adequate IMN doses (45–50 Gy) when IMNs were not included in the clinical target volume (CTV).

## Materials and Methods

### Patient Selection

Patients were eligible if they had undergone PMRT and were newly diagnosed with histologically confirmed invasive stage I, II, or III breast carcinoma. All patients were confirmed to have no clinical or pathological evidence of IMN involvement at the time of diagnosis, and IMNs were not included in the CTV. The institutional research ethics board of Shandong Cancer Hospital and Institute approved this study (SDTHEC201703014), and all methods were performed in accordance with the relevant guidelines and regulations. The requirement to obtain written informed consent from patients was waived due to the retrospective nature of the investigation (retrospective single-institution cohort study).

### Delineation of Target Volume and IMN

The CTV of the chest wall and supraclavicular fossa (SCF) was delineated based on the Radiation Therapy Oncology Group (RTOG) breast cancer contouring atlas (online at: http://www.rtog.org/CoreLab/ContouringAtlases/BreastCancerAtlas.aspx). The chest wall cranial border is variable, depending on contralateral breast size and patient position, and the border is clinically located at the base of the caudal border of the clavicle head. The medial border does not cross midline and is highly variable depending on mastectomy scar. The CTV posterior border was the junction of chest wall muscles and the rib or rib-pleural interface, depending on the T and N stage. The planning target volume (PTV) was generated by the addition of a 5-mm margin to the CTV in all directions. The PTV was limited by 0 mm from the skin, and the amount of lung could be trimmed according to physician discretion. A 5-mm bolus was used over the chest wall. The heart was contoured along with the pericardial sac. The superior aspect was begin at the level of the inferior aspect of the pulmonary artery passing the midline and extend inferiorly to the apex of the heart (online at: http://www.rtog.org/CoreLab/ContouringAtlases/RTOG1106OAR~Atlas.aspx).

Throughout the study, the IMN CTV was defined by the same radiation oncologist. The IMN was also contoured according to the RTOG breast cancer contouring atlas: from the first to third intercostal spaces (ICS1-3) through the topography of the internal thoracic vessels. The IMN PTV (PTV_IMN_) was designed to include an expansion of 5 mm around the IMN CTV. The same contouring atlas was followed to minimize the interobserver variability in the IMNs and achieve the most precise and objective comparison.

### Treatment Planning

The prescription dose was 50 Gy in 25 fractions (2 Gy per fraction) to the PTV, 5 days per week, delivered for 5 weeks. The enrolled patients were treated with one of the three irradiation techniques described below.

#### 3D-CRT

The chest wall was treated with two opposite tangential fields using 6-MV photon beams and an ipsilateral SCF with a single anterior field. The criteria of the 3D-CRT plan ensured that at least 90% of the PTV received the prescription dose.

#### Field-In-Field Forward Intensity-Modulated Radiotherapy (F-IMRT)

The chest wall treatment plan involved the use of a tangential field technique with static multileaf collimator segments, with two parallel-opposed tangential fields using 6-MV photon beams. Two to five segmented fields were manipulated to maintain dose delivery to OARs, such as the ipsilateral lung (IPSL), and the heart within normally accepted tolerances and to reduce the volumes of hot spots in the treatment field. Four to five fields were designed toward the SCF to guarantee dose uniformity. The criteria of the F-IMRT plan ensured that at least 95% of the PTV received the prescription dose.

#### Inverse IMRT (I-IMRT)

The common isocenter was located in the center of the PTV. The tangential field technique was set to the entire PTV, and additional 0° and 40° MLC segments were constructed toward the SCF. The criteria of the I-IMRT plan also ensured that at least 95% of the PTV received the prescription dose. Additional subfields were set to shield the areas of PTV receiving dose >110% of the prescription dose, and keeping the dose delivered to OARs within normally accepted tolerances.

### Anatomy and Technical Factors

Height, body weight, body mass index (BMI), and body surface area (BSA), T- and N-stage were documented in all patients when available from clinical records. In the transverse view of the planning CT images, the number of internal thoracic vessels were counted, and the distance between the most anterior limit of the thoracic vertebrae and the most posterior limit of the sternum in the uppermost level of the inferior vena cava was measured and defined as the thoracic anteroposterior diameter (D_AP_). Thoracic transverse diameter (D_T_) was the greatest horizontal distance of the inner wall of the thorax from the uppermost level of the inferior vena cava. The ratio of D_T_ to D_AP_ was defined as R_T/AP_.

Both CTV and PTV volumes, PTV_IMN_ volume and PTV_IMN_ volume included in PTV (IMNin) were obtained through the Eclipse treatment planning system (TPS) (Eclipse 13.5; Varian Medical Systems, Palo Alto, CA). And the ratio of IMNin to PTV_IMN_ (R_IMNin_) was calculated as R_IMNin_ = IMNin/ PTV_IMN_. As potential factors, CTV and PTV borders, wedge shaped plate, SCF, and irradiation technique were also documented in all patients. Gantry angles ranged from 39 to 60° for the medial fields and from 220 to 253° for the lateral fields for right-sided PTV, and the gantry angles ranged from 300 to 320° for the medial fields and from 115 to 140° for the lateral fields for left-sided PTV. The angle between the medial field gantry angle and horizontal line may also be critical in affecting the IIMN dose and was defined as an incident angle. For right-sided breast cancer patients, this angle is equal to the gantry angles for the medial fields, while for left-sided breast cancer patients, this angle is equal to gantry angle minus 180°.

### Statistical Analysis

Statistical analysis was performed with the SPSS statistical analysis software package. Based on the normality of the distributions, *t*-tests or one-way analysis of variance (ANOVA) was used to assess the statistical significance of the differences between the covariates. Univariate regression analysis and multiple regression analysis were used to assess the relationship between IIMNI dose differences and a set of covariates, such as body weight, BMI, BSA, and radiotherapy technique. All tests were two-sided. Results were regarded as statistically significant when *p* < 0.05.

## Results

### Patients and Treatments

One hundred and thirty-eight breast cancer patients who underwent PMRT between 2012 and 2016 were enrolled in this retrospective study. Table 1 outlines the patient and treatment characteristics. None of the patients received radiotherapy to the ipsilateral IMN.

**Table 1:** Patient characteristics and treatment variables.

**Characteristic**	***n***	**%**
Age (y)		
Minimum	25	18.12
Maximum	74	53.62
Median	47	
Histology		
Invasive ductal carcinoma	134	97.10
Invasive lobular carcinoma	3	2.17
Invasive papillary carcinoma	1	0.72
Tumor location		
Left-sided	73	52.90
Right-sided	65	47.10
T stage		
T0	2	1.45
T1	39	28.26
T2	79	57.25
T3	11	7.97
T4	6	4.35
Tx	1	0.72
N stage		
N0	6	4.35
N1	46	33.33
N2	52	37.68
N3	33	23.91
Nx	1	0.72
Radiotherapy		
3D-CRT	48	34.78
F-IMRT	49	35.51
I-IMRT	41	29.71
PTV		
Chest wall	7	5.07
Chest wall+SCF	131	94.93

*SCF, supraclavicular fossa; 3D-CRT, three-dimensional conformal radiotherapy; F-IMRT, field-in-field forward intensity-modulated radiotherapy; I-IMRT, inverse IMRT*.

### Incidental IMN Dose Coverage

**Figure 1:**
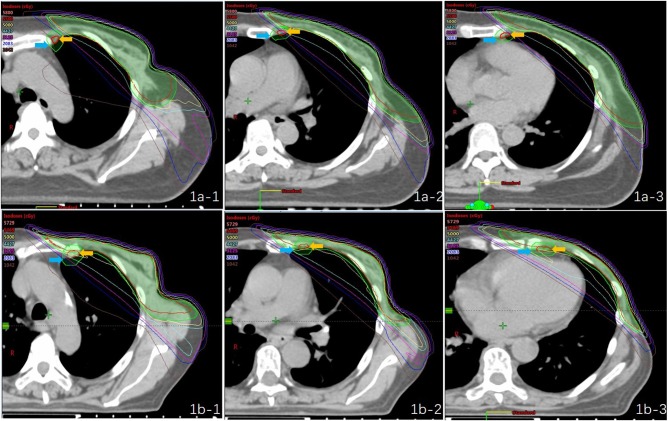
Treatment plans and dose distribution of two “extreme” patients [**(a)** PTVIMN was 2.76 Gy; **(b)** PTVIMN was 50.93 Gy; -1: first intercostal space,−2: second intercostal space,−3: third intercostal space; yellow arrow: the IMN CTV; blue arrow: the IMN PTV].

The mean dose for the PTV_IMN_ was 32.85 Gy for all patients (SD, 9.49 Gy), ranged from 2.76 Gy (Figure 1a) to 50.93 Gy (Figure 1b). There were no significant differences between right breast cancer patients and left breast cancer patients (34. 41 ± 9.14 Gy vs. 32.09 ± 9.68 Gy). Adequate coverage of the PTV_IMN_, defined as ≥45 Gy, was achieved for 10 out of the 138 breast cancer patients with PMRT (7.25%). The clinical and anatomic parameters influencing doses to IMNs are summarized in Table 2. Patients were separated according to IIMNI dose ≥45 Gy and <45 Gy, and body weight, BMI, BSA, and D_T_ were found to be lower in patients with IIMNI dose ≥45 Gy than in patients with IIMN dose <45 Gy. While the higher IIMNI doses were associated with the more IMNin and R_IMNin_. Patient height, D_AP_, R_T/AP_, number of internal thoracic vessels, CTV volume, PTV_IMN_ volume, CTV and PTV border, and gantry angles showed no significant differences between the two groups. There was also no difference in the number of patients using a wedge-shaped plate for patients who underwent 3D-CRT; while 50% of the patients received an IIMNI dose ≥45 Gy using a wedge-shaped plate, only 23.26% of the patients received an IIMNI dose <45 Gy using a wedge-shaped plate.

**Table 2:** Comparison of covariates between patients with and without adequate IMN coverage.

**Characteristic**		**Total**	**≥45 Gy**	**<45 Gy**	***p-*value**
Number of patients		138	10	128	
Cancer laterality	Right	67 (48.55%)	3 (30%)	64 (50%)	0.909
	Left	71 (51.45%)	7 (70%)	64 (50%)	
Height (cm)	Mean	160.16	158.7	160.27	0.441
	SD	5.54	4.79	5.59	
Body weight (kg)	Mean	66	58.6	66.57	0.002
	SD	8.83	4.485	8.84	
BMI	Mean	25.74	23.36	25.93	0.010
	SD	3.30	2.70	3.27	
BSA	Mean	1.67	1.57	1.68	0.007
	SD	0.13	0.05	0.13	
D_AP_ (cm)	Mean	10.01	9.71	10.04	0.438
	SD	1.27	1.49	1.26	
D_T_ (cm)	Mean	23.25	22.71	23.29	0.043
	SD	1.24	0.38	1.28	
R_T/AP_ (%)	Mean	43.21	42.77	43.25	0.796
	SD	6.28	6.84	6.27	
Internal thoracic vessels, *n*	2	128 (92.75%)	9 (90%)	119 (92.97%)	0.728
	3	10 (7.25%)	1 (10%)	9 (7.03%)	
CTV volume (cm^3^)	Mean	436.71	409	438.88	0.622
	SD	138.16	90.2	141.24	
PTV_IMN_ volume (cm^3^)	Mean	29.44	28.92	29.48	0.748
	SD	5.33	4.27	5.43	
IMNin (cm^3^)	Mean	5.61	13.33	5.00	0.000
	SD	4.88	3.54	4.45	
R_IMNin_ (%)	Mean	18.92	46.33	16.78	0.000
	SD	15.93	11.80	14.14	
Irradiation technique, *n*	3D-CRT	48 (34.78%)	5 (50%)	43 (33.59%)	0.112
	F-IMRT	49 (35.51%)	1 (10%)	48 (37.50%)	
	I-IMRT	41 (29.71%)	4 (40%)	37 (28.91)	
SCF, *n*	Yes	131 (94.93%)	10 (100%)	121 (94.53%)	0.449
	No	7 (5.07%)	0 (0)	7 (5.47%)	
Cranial border (CTV), *n*	Clinical reference + second rib insertion	65 (47.10%)	4 (40%)	61 (47.66%)	0.642
	Caudal border of the clavicle head	73 (52.90%)	6 (60%)	67 (52.34%)	
Medial border (CTV), *n*	Midline	33 (23.91%)	5 (50%)	27 (21.26%)	0.190
	Sternal-rib junction	78 (56.52%)	3 (30%)	75 (59.06%)	
	In between	27 (19.57)	2 (20%)	25 (19.68%)	
Posterior border (PTV), *n*	Intrapulmonary	78 (56.52%)	8 (80%)	70 (54.69%)	0.121
	Rib-pleural interface	60 (43.48%)	2 (20%)	58 (45.31%)	
Wedge shaped plate, *n*	Yes	15 (31.25%)	5 (50%)	10 (23.26%)	0.069
	No	33 (68.75%)	5 (50%)	33 (76.74%)	
Incident angles (°)	Mean	52.64	52.3	52.66	0.993

*BMI, body mass index; BSA, body surface area; CTV, clinical target volume; PTV, planning target volume; D_T_, thoracic transverse diameter; D_AP_, thoracic anteroposterior diameter; SCF, supraclavicular fossa; PTV_IMN_, planning target volume of IMN; IMNin, PTV_IMN_ included in PTV*.

### Factors Related to IIMNI Dose Changes

A summary of the factors influencing the IIMNI dose is shown in Table 3. In the univariate analysis, the IIMN dose was significantly higher in patients with lower body weight, BMI, BSA, D_T_, higher R_T/AP_, IMNin, R_IMNin_, and whose PTV posterior border was intrapulmonary. There were no significant relationships observed between the Dmean of the IIMNI dose and the magnitude of patient height, D_AP_, number of internal thoracic vessels, CTV/PTV/PTV_IMN_ volume, CTV/ PTV border, gantry angles, T-stage, N-stage, or the radiation delivery technique for all patients. In multivariate analysis, three variables remained significant; thus, body weight, R_T/AP_, and R_IMNin_ were the correlative factors that affected IIMNI dose (Table 3).

**Table 3:** Univariate and multivariate regression analysis of IIMN dose difference.

**Characteristic**	**Univariate analysis**		**Multivariate analysis**	
	***r***	***p-*value**	**Coefficient (SE)**	***p-*value**
Height	−0.150	0.080		
Body weight	−0.443	0.000	−4.016	0.000
BMI	−0.363	0.000		
BSA	−0.429	0.000		
T stage	0.089	0.301		
N stage	0.130	0.130		
D_AP_	0.074	0.387		
D_T_	−0.407	0.000		
R_T/AP_	0.215	0.011	5.359	0.000
Internal thoracic vessels	0.128	0.134		
CTV volume	−0.091	0.287		
PTV volume	0.004	0.959		
PTV_IMN_ volume	−0.035	0.680		
IMNin	0.734	0.000		
R_IMNin_	0.757	0.000	12.697	0.000
Irradiation technique	0.116	0.175		
SCF	−0.919	0.828		
Cranial border (CTV)	−0.018	0.835		
Medial border (CTV)	−0.110	0.199		
Posterior border (PTV)	−0.286	0.001		
Wedge-shaped plate	0.250	0.087		
Incident angles	−0.091	0.287		

*BMI, body mass index; BSA, body surface area; CTV, clinical target volume; PTV, planning target volume; D_T_, thoracic transverse diameter; D_AP_, thoracic anteroposterior diameter; SCF, supraclavicular fossa; PTV_IMN_, planning target volume of IMN; IMNin, PTV_IMN_ included in PTV*.

## Discussion

Extended resection of the IMNs, which can jeopardize the survival of breast cancer patients even more than the breast cancer itself, has been abandoned (23). IMNI has been shown to have a significant impact on locoregional control, breast cancer mortality, and OS (5–7), but controversies concerning IMNI still exist, mainly because of radiotherapy-induced long-term lung and heart toxicity (8, 24, 25). Though the incidental dose to the IMNs not achieve clinically significant therapeutic levels (26, 27), recent studies have demonstrated that accepting adequate doses during incidental irradiation can result in a clinical benefit (6, 10). By determining the variability in the IIMNI dose coverage with standard chest wall tangential fields using surface anatomy (as determined by a planning CT image) and in patients showing IMN drainage on lymphoscintigraphy, Proulx et al. showed that only 14% and 19% of patients had IMNs completely within the tangent fields (28, 29). Our study found significant variability in individual IIMNI dose for patients who accepted PMRT, and only 7.25% of the patients received a meaningful IIMNI dose when the IMNs were not included in the PTV. This led us to realize that even though low mean doses were being reported, there was significant dose inhomogeneity in the IMNs. The incidental dose to the IMNs may change according to body habitus according to Proulx et al. (28). These authors reported that presternal fat thickness was inversely correlated with IMN inclusion in tangent fields, while thoracic skeletal shape was not associated with IMN inclusion. These studies used the patient presternal fat thickness as a surrogate for body habitus, whereas the present study evaluated the influence of patient height, body weight, BMI, BSA, the thoracic anteroposterior diameter (D_AP_), the thoracic transverse diameter (D_T_), and R_T/AP_ on the IIMNI dose to the patients who accepted PMRT. We postulated that patient anatomy and technical parameters were the potential factors influencing IIMNI dose variance and attempted to determine whether the combination of such influencing factors could potentially indicate a subgroup of patients in whom it would be appropriate to avoid IMN radiotherapy.

According to Sapienza et al. (14) and Arora et al. (15), the predictor for incidental dose to the IMNs is the T-stage and N-stage of the tumor. These authors revealed that advanced N- and T-stage involvement was correlated with higher average doses to IMNs. However, these results are contrary to those of the present study, in which both univariate and multivariate analyses confirmed that the T-stage and N-stage of tumors showed no correlations with the IIMNI dose. This variance may partly be due to the fact that some of the patients enrolled in the previous studies were early breast cancer patients who accepted BCS, and different operative approaches result in variance of presternal fat thickness, which was associated with IMN inclusion (28), and the radiation oncologists considered the patients to be at high risk and adjusted the fields accordingly.

Significantly decreasing the irradiation dose delivered to OARs (lung, heart, and spinal cord), IMRT improves the accuracy of breast cancer radiotherapy (30–32). While the IIMNI dose were consistent, regardless of the irradiation technique (3D-CRT: 33.80 Gy, F-IMRT: 29.65 Gy, and I-IMRT: 32.95 Gy, respectively) (26). Similar to Sapienza et al. (14), we observed that the addition of SCF field irradiation did not significantly increase the Dmean of the IMNs in the PMRT patients. Regardless of the radiotherapy technique, patient anatomy and clinical factors could eventually influence the dose distribution. Previous studies have associated body habitus with nodal volume dose distribution when breast cancer irradiation is administered (20, 33). The results of these present study showed that obese or overweight patients had poorer dose coverage with fixed depth prescription than normal weight patients, and higher doses were associated with the more voluminous (≥1,200 cc) and pendulous breasts. Barry et al. (22) found no direct correlation between BMI and any IMN dosimetric parameter during both free breathing and active breathing control in 50 left-sided breast cancer patients. And this result is consistent with our findings, BMI is not sufficient to predict IIMNI dose, which was insignificant in the multivariate analysis (Table 3).

This analysis showed the higher IIMNI doses were associated with the less body weights and more R_IMNin_. However, only 10 patients received a meaningful incidental irradiation dose during PMRT, and thus, this sample size was insufficient to acquire a more clinically significant difference that would warrant identification of a subgroup of patients for whom it may be more appropriate to avoid IMNI for further management. Additional studies with a larger sample size are needed to provide more continuous up-to-date information.

## Conclusion

The present analysis showed that a small number of breast cancer patients had adequate IIMNI dose coverage for postmastectomy chest wall ± SCF irradiation, which might contribute to controlling IMN micrometastases. The patient body weights, R_T/AP_, and R_IMNin_ was the most important influence factor for IIMNI doses. The risk of avoiding IMNI is different for PMRT patients with different body habitus in breast cancer subpopulations with a high risk of metastatic or even microscopic metastatic IMNs.

## Data Availability Statement

The datasets used and analyzed during the current study are available from the corresponding author on reasonable request.

## Ethics Statement

The studies involving human participants were reviewed and approved by The institutional research ethics board of the Shandong Tumor Hospital Ethics Committee (SDTHEC201703014). Written informed consent for participation was not required for this study in accordance with the national legislation and the institutional requirements. Written informed consent was not obtained from the individual(s) for the publication of any potentially identifiable images or data included in this article.

## Author Contributions

WW and JL participated in the study design, and contributed to the data collection, and draft the manuscript. TS, YZ, MX, and QS participated in the treatment planning. JW, PQ, and XL made important contributions in collecting and analyzing data, and in revising the content. All authors read and approved the final manuscript.

### Conflict of Interest

The authors declare that the research was conducted in the absence of any commercial or financial relationships that could be construed as a potential conflict of interest.

## References

[1] Recht A Edge SB Solin LJ Robinson DS Estabrook A Fine RE. American society of clinical oncology. postmastectomy radiotherapy: clinical practice guidelines of the american society of clinical oncology. J Clin Oncol. (2001) 19:1539–69. 10.1200/JCO.2001.19.5.1539 11230499

[2] Ragaz J Olivotto IA Spinelli JJ Phillips N Jackson SM Wilson KS. Locoregional radiation therapy in patients with high-risk breast cancer receiving adjuvant chemotherapy: 20-year results of the British Columbia randomized trial. J Natl Cancer Inst. (2005) 97:116–26. 10.1093/jnci/djh297 15657341

[3] EBCTCG (Early Breast Cancer Trialists' Collaborative Group) McGale P Taylor C Correa C Cutter D Duane F. Effect of radiotherapy after mastectomy and axillary surgery on 10-year recurrence and 20-year breast cancer mortality: meta-analysis of individual patient data for 8135 women in 22 randomised trials. Lancet. (2014) 383:2127–35. 10.1016/S0140-6736(14)60488-8 24656685 PMC5015598

[4] Recht A Comen EA Fine RE Fleming GF Hardenbergh PH Ho AY. Postmastectomy radiotherapy: an american society of clinical oncology, american society for radiation oncology, and society of surgical oncology focused guideline update. J Clin Oncol. (2016) 34:4431–42. 10.1200/JCO.2016.69.1188 27646947

[5] Thorsen LB Offersen BV Danø H Berg M Jensen I Pedersen AN. DBCG-IMN: a population-based cohort study on the effect of internal mammary node irradiation in early node-positive breast cancer. J Clin Oncol. (2016) 34:314–20. 10.1200/JCO.2015.63.6456 26598752

[6] Poortmans PM Collette S Kirkove C Van Limbergen E Budach V Struikmans H EORTC radiation oncology and breast cancer groups. internal mammary and medial supraclavicular irradiation in breast cancer. N Engl J Med. (2015) 373:317–27. 10.1056/NEJMoa1415369 26200978

[7] Dess RT Liss AL Griffith KA Marsh RB Moran JM Mayo C. Ischemic cardiac events following treatment of the internal mammary nodal region using contemporary radiation planning techniques. Int J Radiat Oncol Biol Phys. (2017) 99:1146–53. 10.1016/j.ijrobp.2017.06.2459 28864405

[8] Taylor CW Wang Z Macaulay E Jagsi R Duane F Darby SC. Exposure of the heart in breast cancer radiation therapy: a systematic review of heart doses published during 2003 to 2013. Int J Radiat Oncol Biol Phys. (2015) 93:845–53. 10.1016/j.ijrobp.2015.07.2292 26530753

[9] Boekel NB Jacobse JN Schaapveld M Hooning MJ Gietema JA Duane FK. Cardiovascular disease incidence after internal mammary chain irradiation and anthracycline-based chemotherapy for breast cancer. Br J Cancer. (2018) 119:408–18. 10.1038/s41416-018-0159-x 30065254 PMC6133926

[10] Xie L Higginson DS Marks LB. Elective regional nodal irradiation in patients with early-stage breast cancer. Semin Radiat Oncol. (2011) 21: 66–78. 10.1016/j.semradonc.2010.08.006 21134656

[11] Giuliano AE McCall L Beitsch P Whitworth PW Blumencranz P Leitch AM Locoregional recurrence after sentinel lymph node dissection with or without axillary dissectionin patients with sentinel lymph node metastases: the American College of Surgeons Oncology Group Z0011 randomized trial. Ann Surg. (2010) 252:426–32. 10.1097/SLA.0b013e3181f08f32 20739842 PMC5593421

[12] Krag DN Anderson SJ Julian TB Brown AM Harlow SP Costantino JP. Sentinel-lymph-node resection compared with conventional axillary-lymph-node dissection in clinically node-negative patients with breast cancer: overall survival findings from the NSABP B-32 randomised phase 3 trial. Lancet Oncol. (2010) 11:927–33. 10.1016/S1470-2045(10)70207-2 20863759 PMC3041644

[13] Kanyilmaz G Aktan M Koc M Demir H Demir LS. Unplanned irradiation of internal mammary lymph nodes in breast cancer. Radiol Med. (2017) 122:405–11. 10.1007/s11547-017-0747-5 28255809

[14] Sapienza LG Chen MJ Gomes MJ Mansur DB. Unintended irradiation of internal mammary chain - Is that enough? Rep Pract Oncol Radiother. (2016) 21:25–30. 10.1016/j.rpor.2015.07.006 26900354 PMC4716404

[15] Arora D1 Frakes J Scott J Opp D Johnson C Song J. Incidental radiation to uninvolved internal mammary lymph nodes in breast cancer. Breast Cancer Res Treat. (2015) 151:365–72. 10.1007/s10549-015-3400-9 25929764

[16] Chung Y Kim JW Shin KH Kim SS Ahn SJ Park W. Dummy run of quality assurance program in a phase 3 randomized trial investigating the role of internal mammary lymph node irradiation in breast cancer patients: Korean Radiation Oncology Group 08-06 study. Int J Radiat Oncol Biol Phys. (2015) 91:419–26. 10.1016/j.ijrobp.2014.10.022 25636764

[17] Leite ET Ugino RT Santana MA Ferreira DV Lopes MR Pelosi EL. Incidental irradiation of internal mammary lymph nodes in breast cancer: conventional two-dimensional radiotherapy versus conformal three-dimensional radiotherapy. Radiol Bras. (2016) 49:170–5. 10.1590/0100-3984.2015.0003 27403017 PMC4938447

[18] Reznik J Cicchetti MG Degaspe B Fitzgerald TJ. Analysis of axillary coverage during tangential radiation therapy to the breast. Int J Radiat Oncol Biol Phys. (2005) 61:163–8. 10.1016/j.ijrobp.2004.04.065 15629607

[19] Kataria T Bisht SS Gupta D Goyal S Jassal K Abhishek A. Incidental radiation to axilla in early breast cancer treated with intensity modulated tangents and comparison with conventional and 3D conformal tangents. Breast. (2013) 22:1125–9. 10.1016/j.breast.2013.07.054 24012148

[20] Aguiar A Gomes Pereira H Azevedo I Gomes L. Evaluation of axillary dose coverage following whole breast radiotherapy: variation with the breast volume and shape. Radiother Oncol. (2015) 114:22–7. 10.1016/j.radonc.2014.10.005 25454171

[21] Lee J Kim SW Son SH. Dosimetric evaluation of incidental irradiation to the axilla during whole breast radiotherapy for patients with left-sided early breast cancer in the IMRT era. Medicine. (2016) 95:e4036. 10.1097/MD.0000000000004036 27368030 PMC4937944

[22] Barry A Rock K Sole C Rahman M Pintilie Lee G. The impact of active breathing control on internal mammary lymph node coverage and normal tissue exposure in breast cancer patients planned for left-sided postmastectomy radiation therapy. Pract Radiat Oncol. (2017) 7:228–33. 10.1016/j.prro.2016.11.010 28139424

[23] Veronesi U Marubini E Mariani L Valagussa P Zucali R The dissection of internal mammary nodes does not improve the survival of breast cancer patients. 30-year results of a randomised trial. Eur J Cancer. (1999) 35:1320–5. 10.1016/S0959-8049(99)00133-1 10658521

[24] Verma V Vicini F Tendulkar RD Khan AJ Wobb J Edwards-Bennett S. Role of internal mammary node radiation as a part of modern breast cancer radiation therapy: a systematic review. Int J Radiat Oncol Biol Phys. (2016) 95:617–31. 10.1016/j.ijrobp.2016.01.058 27131078

[25] Nilsson G Holmberg L Garmo H Terent A Blomqvist C. Radiation to supraclavicular and internal mammary lymph nodes in breast cancer increases the risk of stroke. Br J Cancer. (2009) 100:811–6. 10.1038/sj.bjc.6604902 19259096 PMC2653766

[26] Wang W Zhang Y Xu M Shao Q Sun T Yu T. Postmastectomy radiotherapy using three different techniques: a retrospective evaluation of the incidental dose distribution in the internal mammary nodes. Cancer Manag Res. (2019) 11:1097–106. 10.2147/CMAR.S191047 30774438 PMC6361227

[27] Song Y Yu T Wang W Li J Sun T Qiu P. Dosimetric comparison of incidental radiation to the internal mammary nodes after breast-conserving surgery using 3 techniques-inverse intensity-modulated radiotherapy, field-in-field intensity-modulated radiotherapy, and 3-dimensional conformal radiotherapy: a retrospective clinical study. Medicine. (2019) 98:e17549. 10.1097/MD.0000000000017549 31593136 PMC6799772

[28] Proulx GM Lee RJ Stomper PC. Internal mammary lymph node inclusion in standard tangent breast fields: effects of body habitus. Breast J. (2001) 7:111–6. 10.1046/j.1524-4741.2001.007002111.x 11328318

[29] Hare GB Proulx GM Lamonica DM Stomper PC. Internal mammary lymph node (IMN) coverage by standard radiation tangent fields in patients showing IMN drainage on lymphoscintigraphy: therapeutic implications. Am J Clin Oncol. (2004) 27:274–8. 10.1097/01.coc.0000092596.03967.80 15170147

[30] Wright P Suilamo S Lindholm P Kulmala J. Isocentric integration of intensity-modulated radiotherapy with electron fields improves field junction dose uniformity in postmastectomy radiotherapy. Acta Oncol. (2014) 53:1019–26. 10.3109/0284186X.2014.926027 24975374

[31] Haciislamoglu E Colak F Canyilmaz E Dirican B Gurdalli S Yilmaz AH. Dosimetric comparison of left-sided whole-breast irradiation with 3DCRT, forward-planned IMRT, inverse-planned IMRT, helical tomotherapy, and volumetric arc therapy. Phys Med. (2015) 31:360–7. 10.1016/j.ejmp.2015.02.005 25733372

[32] Schubert LK Gondi V Sengbusch E Westerly DC Soisson ET Paliwal BR. Dosimetric comparison of left-sided whole breast irradiation with 3DCRT, forward-planned IMRT, inverse-planned IMRT, helical tomotherapy, and topotherapy. Radiother Oncol. (2011) 100:241–6. 10.1016/j.radonc.2011.01.004 21316783

[33] Sabater S Gascon M Gutierrez-Perez M Berenguer R Donovan EM Harris EJ. Influence of body habitus on dose parameters of nodal levels III to IV irradiation for breastcancer: comparison of 3 techniques. Med Dosim. (2018) 43:328–33. 10.1016/j.meddos.2017.11.002 29223303

